# Alternative pre-approved and novel therapies for the treatment of anthrax

**DOI:** 10.1186/s12879-016-1951-y

**Published:** 2016-11-03

**Authors:** Breanne M. Head, Ethan Rubinstein, Adrienne F. A. Meyers

**Affiliations:** 1 Department of Medical Microbiology and Infectious Diseases, University of Manitoba, Winnipeg, MB R3E 0J9 Canada; 2 National Laboratory for HIV Immunology, JC Wilt Infectious Disease Research Centre, Public Health Agency of Canada, Winnipeg, Canada; 3 Department of Medical Microbiology, University of Nairobi, Nairobi, Kenya

**Keywords:** *Bacillus anthracis*, Anthrax, Spore, Toxin, Therapeutics, Anti-toxins, Antibiotics

## Abstract

**Background:**

*Bacillus anthracis,* the causative agent of anthrax, is a spore forming and toxin producing rod-shaped bacterium that is classified as a category A bioterror agent. This pathogenic microbe can be transmitted to both animals and humans. Clinical presentation depends on the route of entry (direct contact, ingestion, injection or aerosolization) with symptoms ranging from isolated skin infections to more severe manifestations such as cardiac or pulmonary shock, meningitis, and death. To date, anthrax is treatable if antibiotics are administered promptly and continued for 60 days. However, if treatment is delayed or administered improperly, the patient’s chances of survival are decreased drastically. In addition, antibiotics are ineffective against the harmful anthrax toxins and spores. Therefore, alternative therapeutics are essential. In this review article, we explore and discuss advances that have been made in anthrax therapy with a primary focus on alternative pre-approved and novel antibiotics as well as anti-toxin therapies.

**Methods:**

A literature search was conducted using the University of Manitoba search engine. Using this search engine allowed access to a greater variety of journals/articles that would have otherwise been restricted for general use. In order to be considered for discussion for this review, all articles must have been published later than 2009.

**Results:**

The alternative pre-approved antibiotics demonstrated high efficacy against *B. anthracis* both in vitro and in vivo. In addition, the safety profile and clinical pharmacology of these drugs were already known. Compounds that targeted underexploited bacterial processes (DNA replication, RNA synthesis, and cell division) were also very effective in combatting *B. anthracis*. In addition, these novel compounds prevented bacterial resistance. Targeting *B. anthracis* virulence, more specifically the anthrax toxins, increased the length of which treatment could be administered.

**Conclusions:**

Several novel and pre-existing antibiotics, as well as toxin inhibitors, have shown increasing promise. A combination treatment that targets both bacterial growth and toxin production would be ideal and probably necessary for effectively combatting this armed bacterium.

## Background

*Bacillus anthracis,* the etiological agent of anthrax, is a Gram-positive, sporulating and toxin-producing, rod-shaped bacterium [1, 2]. It is readily found in soil and is responsible for causing disease in livestock including cows, sheep, and goats and wild animals (bison, buffalo) [3]. This pathogen can be transmitted to humans via direct contact, ingestion, aerosolization or injection of vegetative cells or spores resulting in cutaneous, gastrointestinal, inhalational or injectional anthrax, respectively [4]. Cutaneous anthrax (CA), the least severe, albeit the most common form of anthrax, represents approximately 95 % of all reported cases [5, 6]. Clinical presentation of CA often manifests as isolated infections on the face, neck, and arms and is characterized by a black necrotic skin eschar [5, 6]. This form is rarely fatal and can be effectively treated with antibiotics [6]. Gastrointestinal anthrax (GA) is more severe although rare, with no cases having ever been reported in the United States (USA) [7]. Symptoms of GA are considered non-specific (nausea, vomiting, fever, bloody diarrhea and malaise) often resulting in misdiagnosis, leading to treatment delays and high mortality rates of over 50 % [3, 7, 8]. Inhalational anthrax (IA) is the most severe manifestation of anthrax with a mortality rate of up to 90 % if left untreated [9–11]. Similar to GA, this respiratory infection is often misdiagnosed due to non-specific symptoms (fever, cough, fatigue and chest or abdominal pain) [9, 10]. IA rapidly progresses to a fulminant stage of infection resulting in cardiac and pulmonary shock. It can also commonly spread to the brain resulting in meningitis, which is quickly followed by death [9, 10]. The final and most recently identified clinical form of anthrax, known as injectional anthrax, has primarily been associated with heroin drug users in the United Kingdom (UK) and Europe [3]. Since 2009, over 50 cases of injectional anthrax have been reported with a mortality rate of approximately 33 % [3, 12–15].

**Fig. 1:**
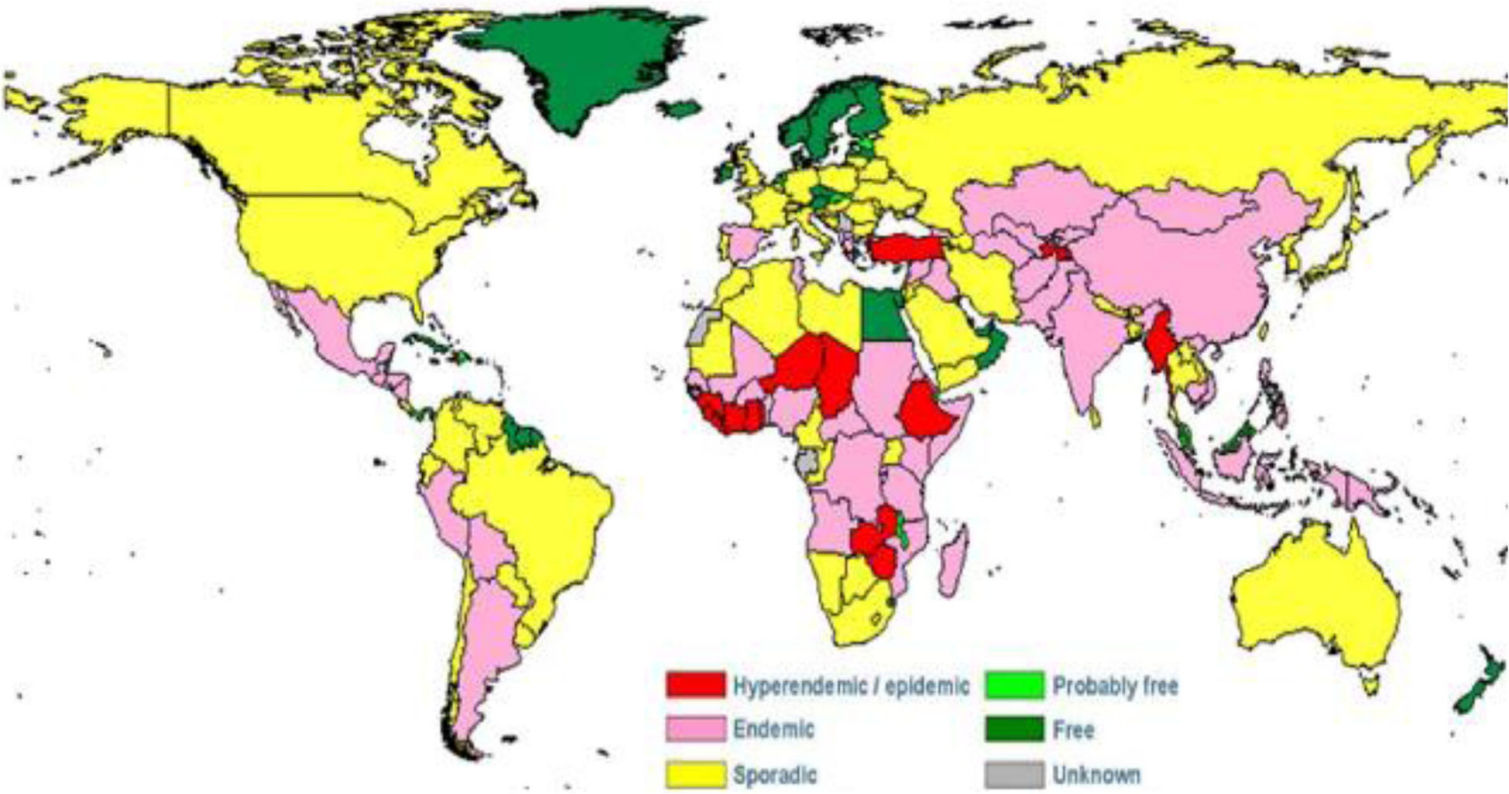
World distribution map of anthrax as determined by the World Health Organization [21]. Reprinted with permission received on April 28^th^, 2015 from the Louisiana State University Department of Veterinary Medicine

Over the last hundred years, there have been numerous documented anthrax outbreaks due to both natural and intentional causes [3, 6, 7, 11, 12, 14–18]. Anthrax is endemic in several developing countries in Africa, Latin America, Eastern Europe and Asia (see Fig. 1) [3, 6, 7, 19–21]. Turkey and Greece are particularly affected due to common practices of animal husbandry, lack of protective measures (such as animal vaccinations) and lack of knowledge about *B. anthracis* [22–24]. Contaminated heroin originating in Afghanistan likely contributed to the 2009 outbreak of injectional anthrax in Europe and the UK possibly due to casing the drug in skins of goats that died from anthrax [25]. In 1979 in Ekaterinburg, Russia (formerly known as Sverdlosk), over 60 people were infected with anthrax due to the accidental release of *B. anthracis* spores from a military microbiology laboratory [18, 26]. Because of this air filter malfunction, 42 residents from the surrounding city perished from IA [26]. In 1993, aerosolized spores were deliberately released by the Aum Shinrikyo cult over Kameido, Japan. However, since the attenuated *B. anthracis* Sterne 34 F2 strain was utilized, no infections were recorded [16]. In 2001, *B. anthracis* Ames strain spores were sent through the USA post office to various news and government offices leading to the exposure of thousands of individuals to anthrax resulting in 22 reported cases (5 deaths) [2, 11, 17].

**Fig. 2:**
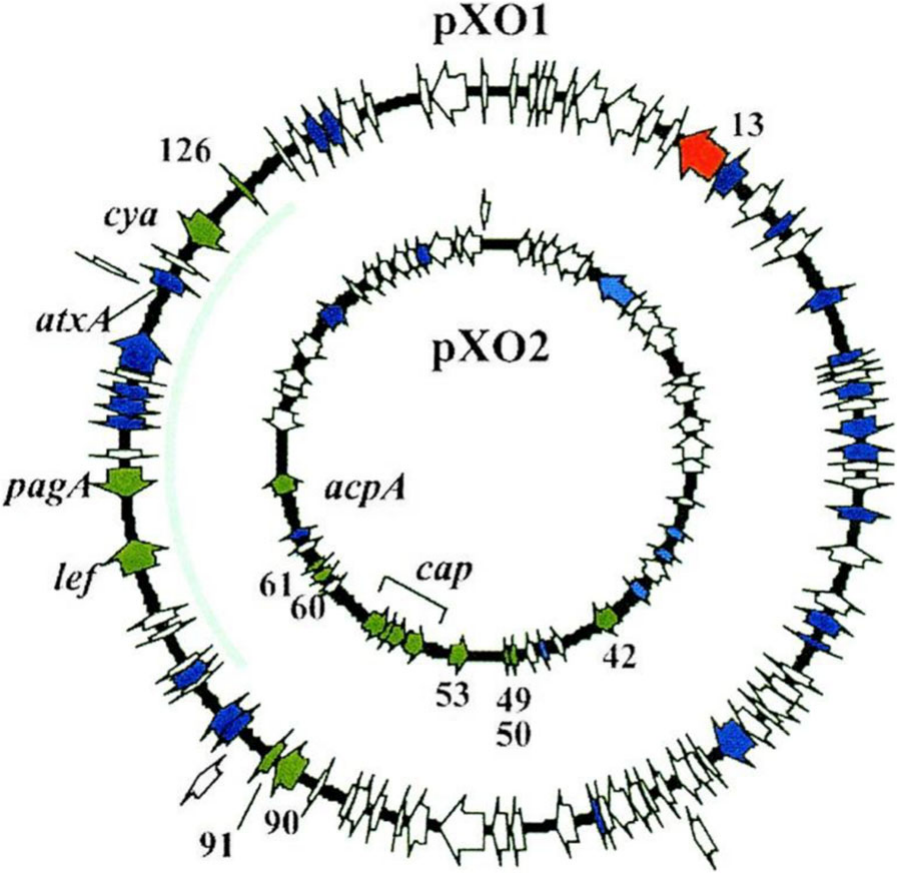
The two plasmids, pXO1 (181.6 kb) and pXO2 (96.2 kb), required for a fully pathogenic *B. anthracis* strain. Image used with permission from Agathe Bourgogne et al [27]

*B. anthracis* pathogenesis is mainly attributed to two large plasmids, pXO2 and pXO1, that are essential for full virulence (see Fig. 2) [27]. pXO2, the smaller of the two plasmids, encodes 80 genes including the *capBCAE* operon responsible for the unique, negatively-charged, poly-γ-D-glutamate capsule that enables host immune evasion and macrophage intracellular survival [1, 27–29]. pXO1 encodes 140 genes including the tripartite exotoxin genes, *pagA*, *lef* and *cya*, which produce the protective antigen (PA), lethal factor (LF), and edema factor (EF), respectively [28]. Once the toxin components are produced, they are secreted by the cell.

**Fig. 3:**
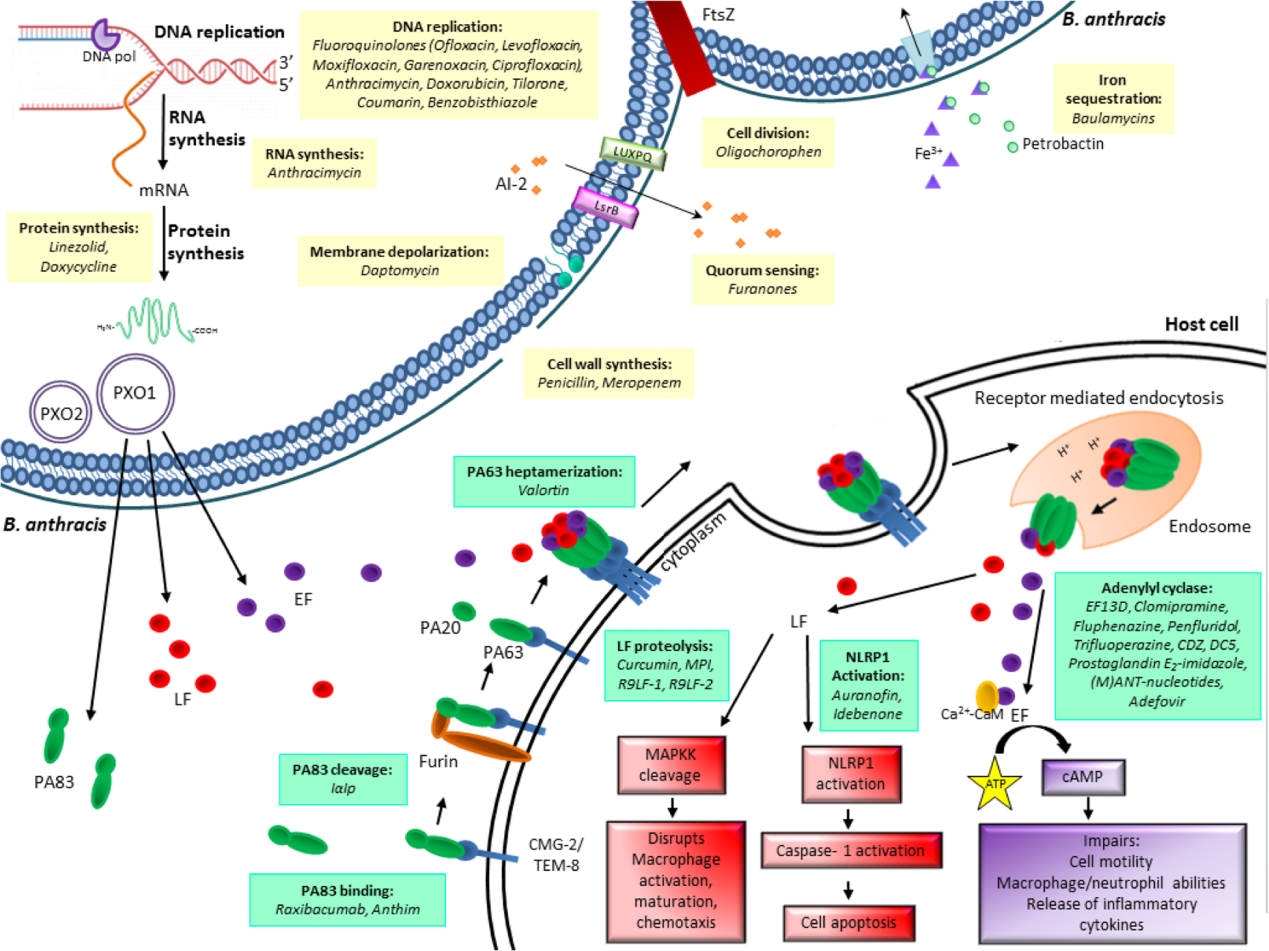
Processes by which antimicrobials (yellow) and anti-toxins (green) interfere with essential *B. anthracis* functions and pathogenesis

PA, the non-enzymatic portion of the toxin, is an 83 kilodalton (kDa) protein (PA83) that facilitates toxin entry into the host cell by binding endothelial cell surface receptors (i.e. tumor endothelial marker-8 [TEM-8] and capillary morphogenesis protein-2 [CMG-2]) throughout the body (see Fig. 3) [30, 31]. Once PA83 is bound to host receptors, it is cleaved by host proteases, namely furin, into two fragments referred to as PA20 (a 20 kDa PA) and PA63 (a 63 kDa PA) [32]. Following the disassociation of PA20, the remaining cell-bound PA63 heptamerizes and binds up to 3 molecules of LF and/or EF to form the lethal toxin (LT) or edema toxin (ET), respectively [33–35]. The toxin complexes are subsequently translocated into the host cell via receptor-mediated endocytosis and delivered to the endosome. Here, the acidic pH induces a conformational change resulting in the release of LF and EF into the cell cytosol where they can exert their enzymatic properties [35, 36].

EF is an 89 kDa calcium and calmodulin (CaM)-dependant adenylate cyclase that catalyzes the reaction of adenosine triphosphate (ATP) to 3, 5′-cyclic adenosine monophosphate (cAMP) [37]. The sudden increase of cAMP prevents apoptosis, cell motility, macrophage and neutrophil abilities as well as impairs the release of several inflammatory cytokines [38–40].

LF is a zinc-dependant metalloproteinase that cleaves and inactivates mitogen-activated protein kinase kinases (MAPKKs) resulting in the disruption of many signalling pathways such as macrophage activation, maturation, and chemotaxis, as well as induces cell death [40, 41]. In addition, in certain rodents, LT also targets NLRP1 (an inflammasome sensor) resulting in the activation of the caspase-1 dependent cell death signalling pathway [42, 43]. This pathway has been studied extensively and is the basis for many LT assays since LT is especially cytotoxic to rodents resulting in rapid macrophages lysis and cell death [42].

Through ET and LT, *B. anthracis* has evolved to target multiple cells throughout the host. In addition to targeting the innate immune system, ET interferes with the adaptive immune system by impairing lymphocyte function ensuring the establishment of the *B. anthracis* infection and future bacterial growth [43, 44]. Furthermore, it targets hepatocytes and intestinal epithelial cells leading to extensive tissue edema [43, 44]. LT primarily targets cardiomyocytes and smooth muscle cells [43–45]. Through liver and cardiovascular impairment, *B. anthracis* is able to impact two vital host systems resulting in lethal vascular collapse.

In order to effectively treat anthrax, prompt recognition and therapeutics are essential. Once the secreted toxins accumulate in the body, antibiotics are rendered ineffective and the patient’s chances of survival are decreased drastically [2, 11, 17]. Once *B. anthracis* is suspected, antibiotic administration should be commenced immediately and continued for 60 days [2]. If clinical symptoms are absent, doxycycline or penicillin G should be administered orally or intravenously (i.v.) at 100 mg twice a day (BID) or 1,200,000 units every 12 h, respectively [46]. Conversely, if clinical symptoms do manifest, an oral or i.v. formulation of 400 mg of ciprofloxacin BID is recommended [47]. Despite this lengthy administration period, current antimicrobials are ineffective against the *B. anthracis* spore. In non-human primates (NHP), spores have been shown to germinate up to 100 days post infection resulting in death rates of up to 30 % [47–50]. Thus, the most promising way to eradicate *B. anthracis* might be to prevent the sporulation process altogether. Studies looking at the effect of the “gold standard” antibiotics on *B. anthracis* sporulation found that treatment with doxycycline (a bacteriostatic, protein synthesis inhibitor) resulted in a predominantly vegetative final population while treatment with the bactericidal ciprofloxacin led to a predominantly spore population [51, 52]. Hence, although doxycycline killed more slowly initially, overall it was considered more effective.

Aside from its inefficiency against spores, the lengthy treatment regimen can also result in a decrease in patient compliance (as seen in 2001 when compliance rates were only 40 %) [53, 54]. Furthermore, the possibility of selection of antibiotic resistant mutants due to high antibiotic usage is a risk since *B. anthracis* resistance has been shown to occur gradually over time [55, 56]. In fact, studies have shown that after 21 subcultures, *B. anthracis* ciprofloxacin sensitivity was decreased from 0.1 to 1.6 mg/l [56]. Likewise, after repeated subculturing with doxcycline, the minimum inhibitory concentration (MIC) was 600 times its initial MIC [57]. Although natural resistance is considered to be low in *B. anthracis,* with no naturally occurring ciprofloxacin or doxycycline resistant strains documented to date, lactamase genes (that cleave lactam drugs i.e. penicillin) have been discovered, albeit in order to be functional, they need to be induced [58]. Induction of these genes results in clinical isolates with MICs as high as 128 g/ml and surveillance has found penicillin resistance in approximately 15 % of reported cases [58, 59]. Additionally, reports have been published showing that penicillin, ciprofloxacin and doxycycline resistant strains can be easily bioengineered [55, 60]. Therefore, in the event of a bioterrorist attack, it may be possible that the microorganism being used might be resistant to first-line antibiotic therapy (i.e. penicillin, doxycycline, or ciprofloxacin).

In addition to antibiotics, under certain specific circumstances (i.e. not generally across the population) vaccination is also recommended to aid in the development of an active immune response and to prevent infection with *B. anthracis*. There are two anthrax vaccines that are currently licensed for use: Anthrax Vaccine Precipitated (AVP) which is licensed in the UK and Anthrax Vaccine Absorbed (AVA) which is licensed in the USA. AVP, first licensed in 1979, is a PA-based vaccine made from an avirulent *B. anthracis* Sterne 34 F2 strain and requires 4 intramuscular doses over a period of 32 weeks [61]. AVA or Biothrax (Emergent BioDefense Operations Lansing LLC), a cell-free PA-based vaccine made from the V770-NP1-R *B. anthracis* strain, contains an aluminum adjuvant and is part of the USA strategic national stockpile [7, 62]. A subcutaneous (SC) dose of 0.5 mL of AVA must be administered as five doses at 0 and 4 weeks and at 6, 8 and 12 months to be effective as pre-exposure prophylaxis [63, 64]. Although it is primarily used for pre-exposure prophylaxis in high-risk populations such as military personnel, researchers or veterinarians, AVA has also been shown to be effective in post-exposure situations [62, 65, 66].

Aside from requiring multiple doses, there are many other points to be considered with the current anthrax vaccine. Specifically, AVA has varying amounts of PA per batch, and a limited shelf life (stocks must be replaced every 4 years) [67–69]. Although it is considered safe, adult patients have reported some side effects (lymphadenopathy, immune system disorders, tremor, ulnar nerve neuropathy, as well as musculoskeletal, connective tissue and bone disorders) [64]. Also, the safety of AVA in children less than 18 years of age is currently unknown. In addition, in a 2008 observational study it was found that pregnant USA military women that had been vaccinated with AVA during their first trimester had slightly elevated rates of birth defects compared to non-vaccinated pregnant women. Therefore, AVA is not recommended for pregnant women or people under 18 years of age unless all other options have been exhausted [70].

Furthermore, although PA can elicit a humoral immune response, it is limited in its ability to promote long-lasting immunity (due to a declining anti-PA response over time) [64, 71]. Storage of both the vaccine and antibiotics in such large quantities along with the requirement for constant patient monitoring make these strategies less practical. Consequently, treatment following a scenario of a biological anthrax attack where a large number of individuals are exposed would not be feasible using the current treatment regimens.

In this review article, we explore and discuss advances that have been made in anthrax therapeutics with a primary focus on alternative pre-approved and novel antibiotics as well as anti-toxin therapies that could be useful in the event of a bioterror attack.

## Qualifications for new drugs for the treatment of B. Anthracis infections

Since *B. anthracis* is primarily an agent of bioterrorism, naturally occurring human infections are infrequent. Moreover, due to the high morbidity and mortality associated with anthrax, it would be unethical to perform clinical studies in humans. In the case of bioterrorism agents, the Food and Drug Administration (FDA) may approve a drug based on the Animal Efficacy Rule which states that a drug may receive approval for the treatment of IA if its efficacy has been demonstrated in more than one animal model as long as the models serve as a reasonable substitute for humans [72]. Furthermore, pharmacokinetic studies must be conducted in animals and humans allowing proper dose selection and the mechanisms of drug toxicity must be relatively well understood. Finally, it is expected that the drug will have a favorable endpoint and will improve host survival [73].

## Antibiotics currently approved for the treatment of other bacterial diseases

It is often recommended that the medication being evaluated already be approved for the treatment of other illnesses since their safety profile and clinical pharmacology will already be known. Doxycycline and penicillin G, two antibiotics that have recently been approved for their use in the treatment of IA, had been on the market for upwards of 50 years and had been used to treat over 100 million patients in the USA prior to their approval for anthrax therapy [73].

Second and third generation fluoroquinolones, such as ofloxacin, levofloxacin, and moxifloxacin, have already been approved by the USA FDA under the respective names of Floxin (Ortho-McNeil Pharmaceutical), Levaquin (Janssen Incorporated), and Avelox (Bayer HealthCare Pharmaceuticals) for the treatment of various respiratory and skin infections [74–76]. These fluoroquinolones are bactericidal against a broad spectrum of microorganisms and function by inhibiting the bacterial DNA gyrase and topoisomerase IV (Fig. 3) [75, 77, 78]. Fluoroquinolone resistance has been assessed and although spontaneous in vitro resistance is rare (10^−9^–10^−10^), bioengineering of resistant strains in a laboratory setting is possible and should be taken into consideration [75]. Athamna et al*.* investigated the ability of the *B. anthracis* ST-1 and Sterne strains to develop resistance to ciprofloxacin, ofloxacin, levofloxacin, moxifloxacin and garenoxacin [55]. Within 10 passages, resistance was recorded for all quinolones and after 18 passages, all of the MIC increased from 0.03 to 8 mg/L with the exception of garenoxacin which increased from 0.015 to 0.5 mg/L for the ST-1 strain. Cross-resistance among the fluoroquinolones was also demonstrated; however, being resistant to one fluoroquinolone did not necessarily indicate resistance to all others [55].

Levofloxacin, an isomer of ofloxacin, has demonstrated improved in vitro potency and reduced toxicity compared to its second generation precursor [75]. In 2008, levofloxacin, or more specifically Levaquin (Janssen Incorporation), received approval by the FDA as an alternative therapeutic for the treatment of IA [75]. A 60-day regimen of Levaquin should be administered to both adult and pediatric patients. However, adults and children weighing > 50 kg should be given 500 mg/kg every day (QD) while children weighing < 50 kg should be administered 8 mg/kg BID [75, 79]. Side effects of Levaquin have only been assessed in adult populations and are similar to those of other fluoroquinolones (tendon rupture and tendinopathy, peripheral neuropathy, arthralgia, myalgia, dermatologic reactions, thrombocytopenia, and interstitial nephritis) [75]. In addition, in some juvenile animal studies quinolones have been associated with osteochondrosis [74, 80]. Although the safety profile of using levofloxacin long term (for example in a 60-day regimen) is still currently unknown, due to the gravity of IA and to the current lack of approved alternative antibiotics, the benefits of using a fluoroquinolone such as levofloxacin greatly out-weigh the risks.

Moxifloxacin, another third generation quinolone, has also been assayed for its effectiveness against *B. anthracis*. In a recent study, a hollow-fiber pharmacodynamic model (IPDM) was used to compare the efficacies of moxifloxacin, linezolid and meropenem to the currently prescribed antibiotics in killing the spore forming Sterne and non-spore forming CR4 *B. anthracis* strains [51]. Against the CR4 strain, meropenem killed the fastest followed by the fluoroquinolones (moxifloxacin and ciprofloxacin), with doxycycline and linezolid exhibiting the slowest kill rate. Heat-shock studies demonstrated that the bacterial populations exposed to bactericidal antibiotics (fluoroquinolones and meropenem) consisted primarily of spores while populations treated with doxycycline and linezolid consisted primarily of replicating bacteria. A possible reason for the latter could be because linezolid and doxycycline are protein synthesis inhibitors and may have prevented the population from converting into the spore form [73, 81]. Although these bacteriostatic antibiotics kill at a slower rate, their potential ability to increase the window of antibiotic exposure due to the prevention of spore formation may result in better overall rate of clearance of the total population. These findings were corroborated by another group who compared the efficacy of linezolid to ciprofloxacin in treating *B. anthracis* Sterne strain infections in an IPDM [52]. Their study demonstrated that a dose of 600 mg of linezolid was sufficient to prevent vegetative *B. anthracis* from converting to spores while a 500 mg dose of ciprofloxacin BID was not [52].

To date, linezolid has not been approved by the FDA for the treatment of *B. anthracis* infections. However, it has been approved for the treatment of a variety of other Gram-positive microorganisms including methicillin-resistant *Staphylococcus aureus* (MRSA) and multidrug resistant *Streptococcus pneumonia* under the name Zyvox (Pfizer Canada Inc) [82]. Linezolid is a bacteriostatic oxazolidinone that inhibits bacterial protein synthesis by preventing the formation of the 70S ribosomal complex [83]. Since linezolid has such a unique mode of action, cross resistance with other antibiotic classes is unlikely and has not yet been observed. Thus, linezolid is an attractive therapeutic for penicillin or fluoroquinolone resistant bioterrorism agents. In vitro linezolid resistance occurs at a frequency of approximately 10^−10^ and has been associated with point mutations in the bacterial 23S rRNA gene [82]. Studies have shown that linezolid can reduce the production of the *S. aureus toxin,* toxic shock syndrome toxin-1 [84]. Since linezolid is a protein synthesis inhibitor that can decrease toxin production in other Gram-positive organisms it is reasonable to consider that it may also prevent *B. anthracis* toxin production*.* Louie et al*.* looked at the effect of ciprofloxacin and linezolid on *B. anthracis* PA production at various times throughout a 10-day experiment [52]. They detected PA in the control after 3 h and in levofloxacin from 3 to 8 h. However, no PA was observed with linezolid treatment [52]. In a similar manner, linezolid has also been found to prevent spore production while non-protein synthesis inhibitors, such as moxifloxacin and meropenem, could not [51]. These findings support the hypothesis that a protein synthesis inhibitor (i.e. linezolid) may be a practical way to prevent the deleterious effects of the anthrax toxins while preventing sporulation. Studies looking at resistance to, and the pharmacodynamics of, linezolid have found that a pharmacodynamically optimized regimen of 700 mg QD did not lead to resistance and was just as effective at killing as the clinically prescribed linezolid (600 mg BID) and gold standard ciprofloxacin (500 mg BID) [85]. Not only does this dosing regimen decrease the total dosage patients would be exposed to, but it would also aid in patient compliance by decreasing the dose frequency from BID to QD making it more feasible and more cost effective.

Aside from being a protein synthesis inhibitor, linezolid also has excellent bioavailability and is available in both oral and i.v. formulations [52, 82, 85, 86]. Conversely, one drawback to linezolid is that it is quite costly. The average price of medication for one patient for one day costs approximately $140 Canadian dollars (CAD) which is much higher than levofloxacin ($2 CAD), ciprofloxacin ($2.50 CAD), and doxycycline ($3 CAD) combined making linezolid undesirable if a 60-day regimen is required [87–90]. In addition, although linezolid is considered relatively safe when used for a short term (<2 weeks), side effects such as peripheral neuropathy, thrombocytopenia, and neutropenia have been associated with long term use (>28 days) [86, 91–94]. This toxicity may, however, be reduced by modifying the currently acceptable linezolid regimen to the suggested pharmacodynamic dosage schedule suggested by Louie et al*.* [85]. By the same token, linezolid could be used initially in the event of a bioterror attack and once susceptibilities of the agent have been determined patients could switch to alternative antibiotics such as ciprofloxacin, penicillin or doxycycline.

A newer class of antibiotic, the cyclic lipopeptides, and more specifically daptomycin, has recently gained more interest as a possible surrogate for anthrax therapy. This agent binds to the bacterial membrane depolarizing the membrane potential resulting in DNA, RNA and protein synthesis inhibition ultimately leading to cell death [95–97]. This unique mechanism of action sets daptomycin apart from other antibiotic classes and to date, no resistant isolates have been documented. Currently, cyclic lipopeptides are only approved for the treatment of antimicrobial resistant Gram-positive microorganisms like those of the *staphylococcus*, *streptococcus* and *enterococcus* genera [95]. However, studies have demonstrated that this class is also efficacious against *B. anthracis *in vitro [95, 96]. One study in particular found that a 21-day treatment of daptomycin (SC injection of 50 mg/kg BID) was as effective as a 21-day treatment of ciprofloxacin (intraperitoneal [IP] injection of 30 mg/kg of body weight BID) in treating *B. anthracis* Ames strain infected mice with both treatments yielding survival rates of 90 % [96]. Moreover, no bacteria (vegetative or spores) were cultivable from the lung, spleen and mediastinum samples collected from all of the surviving mice [96]. This mouse inhalational study suggests daptomycin may be a potential candidate as an alternative therapeutic and further in vivo* B. anthracis* studies are warranted.

Several studies have evaluated the effect of combination therapy in order to further increase the rate of bacterial killing and to circumvent bacterial resistance [98, 99]. The combination of rifampin and clindamycin and the combination of telithromycin and amoxicillin have been shown to work synergistically against the *B. anthracis* Sterne strain [98]. Other therapies containing a combination of either ciprofloxacin or tetracycline with clindamycin, rifampin, or linezolid have demonstrated indifference while combinations containing penicillin were found to be antagonistic [98]. Similarly, when combined, linezolid and levofloxacin have been shown to behave antagonistically and resulted in decreased *B. anthracis* killing [100]. Due to the alarming increase in antibiotic resistance and the resilience of *B. anthracis*, further research determining effective combination therapies is merited and will be invaluable.

## Exploiting novel targets for antibiotic development

With an increase in antimicrobial resistance, there is a greater need for the development of newer classes of antimicrobials. Studying essential bacterial processes such as DNA replication, RNA synthesis, and cell division that are currently underexploited would potentially broaden our arsenal against this microbe by leading to new and innovative antibiotics for which there is no pre-existing bacterial resistance mechanism.

Targeting bacterial DNA replication has been successful in the past; however, specifically targeting the bacterial DNA helicase or primase are relatively new ideas [2, 101–103]. Through the use of high-throughput screening, several coumarin-based helicase inhibitors were identified for their efficacy against *B. anthracis* and *S. aureus* [104]. Chemical optimization of these inhibitors led to the discovery of two potent biphenyl coumarin compounds (20 and 22) with half maximal inhibitory concentration (IC_50_) values of 3 and 1 μM (against both microorganisms) that worked non-competitively when inhibiting DNA helicase [105]. In contrast, benzobisthiazole helicase inhibitors and their derivatives work in a competitive manner with DNA and ATP substrates by binding to the helicase active site [106]. Compound 59, the most potent helicase inhibitor identified to date, has a high selectivity index (greater than 500), no observable cytotoxicity, and inhibits *B. anthracis* with an IC_50_ of 0.2 μM [106].

In a recently reported dose–response assay, doxorubicin (an interferon inhibitor), and tilorone (an interferon inducer) demonstrated low micromolar inhibitory activities towards the *B. anthracis* 34 F2 Sterne strain primase DnaG [107]. One challenge for making DnaG inhibitors is that in order to be effective these compounds must be able to access the bacterial cytoplasm. Doxorubicin was able to traverse the bacterial envelope and exert its bacteriostatic effects on the 34 F2 Sterne strain with a MIC of 6.6 μM; however, tilorone could not [107]. Since DnaG is an essential enzyme for chromosomal DNA replication, is moderately conserved among bacterial species (30 % sequence identity between *Bacillus* and *Mycobacterium*), and shares very low sequence similarity to the eukaryotic topoisomerase II, targeting this enzyme could lead to a novel inhibitor with high species specificity [107–109].

Anthracimycin, another inhibitor that targets the DNA replication process, is a structurally unique compound composed of a rare combination of 14-and 6-member rings and is produced by a marine-derived actinomycete [110]. This tricarbocyclic metabolite has demonstrated good potency against the *B. anthracis* UM23C1-1 strain with a MIC of 0.031 μg/mL as well as inhibitory activities against other Gram-positive microorganisms (staphylococci, enterococci, streptococci) [110]. Chlorination of anthracimycin leads to improved bioactivity against a broader spectrum of microbes (including Gram-negatives) while retaining significant potency against *B. anthracis* (MIC of 0.0625 μg/mL) [110]. Although the exact mechanism of action is yet to be elucidated, it is thought to be a DNA and RNA synthesis inhibitor. Furthermore, it has also been suggested that anthracimycin works synergistically with the cathelicidin, LL-37, making microorganisms more sensitive to cathelicidin-mediated killing [111, 112]. LL-37 plays an important part in the host immune response especially in the recruitment of neutrophils, monocytes, and T cells and has been shown to play a role in containing the *B. anthracis* infection [113–115]. Having an antibiotic that works synergistically with a host cationic peptide may increase the initial immune response when exposed to *B. anthracis*. Although in vivo studies looking at the effect of anthracimycin of *B. anthracis* have yet to be carried out, one study looking at the effect of this novel compound on MRSA-infected CD1 mice found that anthracimycin retained high potency in vivo and was well tolerated [111]. Due to its broad spectrum abilities, high potency, and possible host synergy*,* anthracimycin and its derivatives represent a potential new and unique class of antibiotics. Further studies are required to determine the exact mechanism of action by which this compound exerts its effects as well as safety, resistance and appropriate dosing.

Aside from hindering bacterial DNA replication, targeting cell-to-cell cross-talk or other important cell processes (nutrient acquisition or cell division) have also proven to be invaluable [116–118]. The compound 3Z1, which is a FtsZ-targeting oligoclorophen, was recently explored as a clinical agent and was found to have a MIC of 320 nM against the *B. anthracis* Sterne strain (comparable to tetracycline and penicillin G) [119]. Aside from being a potent antimicrobial, developing bacterial resistance to 3Z1 may also be difficult since FtsZ is a conserved protein essential for bacterial cell division [120–122]. Indeed, when studies looked at resistance they found that the *B. anthracis* Sterne 7702 strain developed resistance at a much lower rate to the FtsZ inhibitor, 3Z1, compared to rifampin (4.34x10^10^ and 2.65x10^9^ per generation, respectively) [117].

Baulamycins, produced by *Streptomyces tempisquensis*, target bacterial siderophore synthesis genes necessary for iron sequestration important for growth and survival in iron deficient environments [116]. *B. anthracis* produces an iron scavenger named petrobactin, which is synthesized by a nonribosomal peptide synthetase independent siderophore synthetase, AsbA [118, 123]. Baulamycins A and B inhibited AsbA with an in vitro IC_50_ of 180 μM and 200 μM, respectively, and demonstrated good cell solubility [118, 124–126]. Moreover, these compounds exhibited broad spectrum activities against MRSA, *Escherichia coli*, and *Shigella flexneri*. Further studies are necessary to improve potency and target selectivity; however, baulamycins show great potential.

Targeting the bacterial quorum sensing mechanism which co-ordinates many behaviors (colonization, persistence and often virulence) has been shown to attenuate other pathogenic bacteria [126]. Many *Bacillus*, including *B. anthracis,* synthesize small autoinducers that allow the co-ordination of the toxin genes and cell growth. A study by Jones et al*.* looked at the effect of (5Z)-4-bromo-5-(bromo-methylene)-3-butyl-2(5H)-furanone and several furanone derivatives on the *B. anthracis* autoinducer, Al-2 [116]. Not only did the furanones inhibit log-phase growth on multiple *B. anthracis* strains when added 3 h post-inoculation, but they also significantly reduced toxin gene expression. Moreover, these naturally synthesized furanones have been shown to be stable under storage conditions [116]. Although quorum sensing systems have the potential to lead to promising new therapeutics, little work has been collected on their toxicity in animals; therefore further analysis is still required.

## Anti-toxin therapies

Currently, the main priority for treating many illnesses is to eliminate the replicating bacteria. However, in the case of anthrax, preventing the effects of the toxins is equally important when combatting late stage disease. Throughout the last decade, extensive research has been conducted looking at *B. anthracis* anti-toxins in order to find cheaper, more stable and immunogenic molecules, albeit very few anti-toxins are currently approved for anthrax treatment. Toxin inhibitors can target several steps in the toxin entry process, which include: (i) PA83 binding to host receptor, (ii) PA83 cleavage, (iii) PA83 heptamerization, (iv) LF or EF binding, (v) LF proteolysis, (vi) LF inflammasome activation, and (vii) EF adenylyl cyclase activity (Fig. 3). Since PA is essential for toxin entry and is a component of both ET and LT, many studies have focused on targeting this component (for a full review see Chen, Moayeri, and Purcell [127]). Raxibacumab (Abthrax; GlaxoSmithKline), a human IgG1 monoclonal antibody (mAb) against PA, received FDA approval in 2012 for treating anthrax based on the Animal Efficacy Rule [128–130]. It binds PA with an affinity of 2.78 nM and prevents PA-receptor binding [128]. It is recommended for treating adult and pediatric patients with IA and should be administered in combination with antibacterial drugs. Indeed, when combined with ciprofloxacin, raxibacumab demonstrated potency and did not affect ciprofloxacin function [130]. In addition, it is recommended for IA prophylaxis if alternative therapies are not unavailable [130]. In adults, a 40 mg/kg dose of Raxibacumab should be diluted in 0.9 % Sodium Chloride, USP to a final volume of 250 mL then administered as a single i.v. dosage over 2 h and 15 min. [128, 130] To reduce the risk of reaction, 25–50 mg of diphenhydramine should be administered within 1 h of Raxibacumab. Common adverse reactions include rash, pain in extremity, pruritus, and somnolence [130]. To date, Raxibacumab is the best anti-PA option available; however, there is still opportunity for improvement. This anti-PA mAb cannot cross the blood brain barrier and is not antibacterial; therefore it cannot prevent or treat meningitis (often a consequence of late stage anthrax). [130] In addition, for storage, this medication must be kept refrigerated (2 to 8 °C) and should not be exposed to light. Furthermore, modifying the current route of administration from i.v. to either SC or intramuscular would be more desirable in the event of a bioterror attack.

Anthim (Elusys Therapeutics), a humanized anti-PA mAb that also targets PA-receptor binding, binds PA with an affinity of 0.33 nM [127]. As mentioned by Chen et al*.*, this drug has received Fast-track and orphan drug status since it has demonstrated efficacy in both pre and post-exposure situations in various animal models [127]. In studies where Anthim has been co-administered with levofloxacin, ciprofloxacin or doxycycline, IA-infected animals demonstrated higher survival outcomes compared to solo antibacterial therapy [127, 129]. In fact, in 2016, Anthim received FDA approval as an alternative treatment for adult and pediatric patients with IA and is recommended in combination with antibacterial drugs (ciprofloxacin) when alternatives are unavailable [131]. Due to hypersensitivity reactions and anaphylaxis, Anthim is only recommended if its benefit outweighs the risk. Similar to Raxibacumab, patients must be pre-medicated with diphenhydramine and Anthim must be diluted in Sodium Chloride prior to use. Medication should be administered as a single i.v. dose of 16 mg/kg over 1 h and 30 min. In addition, Anthim does not have antibacterial activity, cannot cross the blood–brain barrier and requires storage in a dark, refrigerated (2 to 8 °C) area.

Targeting the next step in the toxin entry process, PA83 cleavage by Furin, has shown some merit in several studies [32, 132, 133]. Inter-α inhibitor protein (IαIp), a human serine protease, inhibits furin with good efficacy resulting in post-exposure protection in *B. anthracis* Sterne 34 F2-infected AJ mice when combined with moxifloxacin [132]. In addition, treatment with this combination led to normal liver and spleen histopathology with no bacilli present. Since IαIp have been shown to be effective in murine models, the next step is to determine their efficacy in larger IA animal models. Some IαIp, like urinary trypsin inhibitors, have already demonstrated their safety in clinical trials and their potential for the treatment of anthrax disease seems promising [134, 135].

Another PA process that can be targeted is the formation of PA heptamers which enables LF and EF entry to the cell. Valortin (PharmAthene/Medarex) is a fully human anti-PA mAb generated from transgenic mice that interrupts PA heptamerization. This inhibitor has demonstrated prophylactic efficacy in rabbits and monkeys and has obtained Fast-Track, Orphan drug status [127].

As a result of more knowledge and understanding of both EF and LF structure and function, a variety of novel, small molecule inhibitors, antibodies and other drug-like, anti-toxins have been discovered. Curcumin, the active ingredient in turmeric spice (Curcuma longa), is often used in traditional medicine and has demonstrated beneficial activities in combating cancers, inflammatory diseases and anthrax [136–139]. Of late, curcumin has been shown to inhibit metalloproteinases (including LF) by binding to the zinc in their active site [138–141]. Furthermore, chemically modified curcumin has demonstrated improved solubility, stability, and bioavailability, with similar potency and less toxicity [138, 139]. Other lethal factor inhibitors (LFI) that contain a zinc chelating group are R9LF-1, R9LF-2 and modified peptidomimetic inhibitors (MPI) [142–145]. Both R9LF-1 and R9LF-2 are stable in solution and are efficient at inhibiting LF in kinetic assays. However, in a longer murine macrophage assay, the stability of R9LF-1 decreased drastically while R9LF-2 had better stability and macrophage protection. MPI containing portions of either BI-MFM3 (Cengent Therapeutics Incorporated) or L915 (Merck Research Laboratories) have demonstrated high binding abilities to both the LF substrate-binding groove and the catalytic zinc-binding site leading to good LF inhibition.

Aside from targeting LF’s proteolytic ability, studies have also looked at inhibiting LF’s ability to activate the NLRP1 inflammasome (i.e. caspase-mediated apoptosis) [146–149]. Both Auranofin (an organogold compound with anti-inflammatory abilities) and Idebenone (a benzoquinone that has previously been used in Alzheimer’s patients) are speculated to interfere with inflammasome activation. Specifically, Auranofin inhibits LT-mediated caspase-1 activation and catalytic activity while Idebenone inhibits the voltage-gated potassium channels. When combined, these two compounds work synergistically to strongly inhibit the LT activity of *B. anthracis* [146–149].

Although majority of studies have focused on PA and LF inhibition, significant research into EF inhibition has also been conducted (reviewed extensively in [150]). EF inhibition can occur through different ways including targeting the adenylyl cyclase, substrate binding, and allosteric sites. To date, the most promising anti-EF molecule is EF13D, a chimeric chimpanzee/human mAb that neutralizes EF with a very high affinity of 0.05–0.12 nM [127]. Studies have shown that EF13D prevents edema formation as well as rescues ET-challenged mice. It functions by binding CaM and can also displace pre-bound CaM from EF.

Since CaM stimulates EF catalytic activity, certain studies have targeted CaM and the CaM-target interaction [37, 151–153]. Several well-known, potent CaM-inhibitors that have already been discovered and produced for their use in other illnesses, such as depression and psychosis, have demonstrated good efficacy against EF [151, 154–157]. Clomipramine (antidepressant), fluphenazine (antipsychotic), penfluridol (antipsychotic), and trifluoperazine (antipsychotic), were able to inhibit EF by 20 %, 30 %, 45 % and 40 %, respectively. In addition, Calmidazolium chloride (CDZ) was able to abolish EF activity all together [151]. Interestingly, CDZ inhibits EF through an allosteric mechanism (while the other EF inhibitors directly target the CaM-EF binding region) [158]. Unfortunately, CDZ often affects unintentional targets [151]. In a like manner, P-site inhibitors (such as N-methyl anthraniloyl-nucleotides [(M)ANT-nucleotides] and adefovir), that function by targeting the adenylyl cyclase catalytic site, are non-selective between EF and mammalian adenylyl cyclases. Therefore, to date, have not been clinically useful.

An adenylyl cyclase inhibitor that has demonstrated good potential as an EF inhibitor is the fluorine-based compound, DC5 [159, 160]. This compound can inhibit EF with a more potent IC_50_ than prostaglandin E_2_-imidazole (a previously described front-runner EF inhibitor). In addition, it can prevent toxin-induced cAMP accumulation from both enterotoxinogenic *E. coli* and *B. anthracis* [159, 160]. Moreover, through modification of its aromatic group, DC5 derivatives have become more soluble and less toxic (but equally potent) compared to their parent compound.

Since the EF catalytic site has demonstrated close similarity to other bacterial adenylyl cyclases, such as the heat-labile toxin of enterotoxinogenic *E. coli* and the cholera toxin, it may be possible to synthesize EF inhibitors with broad spectrum activity [159, 160]. However, since bacterial and mammalian adenylyl cyclase catalytic sites also share homology, constructing highly selective and potent EF inhibitors may be difficult [150]. Therefore, research looking at targeting EF allosteric sites is also recommended, albeit caution is recommended since targeting allosteric sites, as seen with CDZ, can have off-target effects [151]. Collectively, studies have demonstrated how problematic it has been to create a soluble, highly selective, and potent EF inhibitor.

It is known that combination therapy can have considerable benefits. Particularly, combining toxin inhibitors with antibiotics has proven to be a valuable way to combat several infections from *Pseudomonas* spp, *klebsiella* spp and *B. anthracis* [127, 129–131, 161–164]. Indeed, in a study by Karginov and colleagues, solo treatment with ciprofloxacin was only able to rescue 50 % of the Sterne-infected mice while the combination of ciprofloxacin and anti-PA antibodies was able to rescue more than 90 % [99]. These studies, and others, reiterate the fact that combination therapy may be the most promising means for combatting *B. anthracis*.

## Conclusions

Although *B. anthracis* has been a microorganism of high interest for many years, anthrax still remains a dangerous disease that is often untreatable. A great deal of progress has been made in anthrax therapies with many novel antibiotics and toxin inhibitors showing great potential. Utilizing antibiotics that have already been approved for the treatment of other bacterial infections may prove to be an asset in treating anthrax. Furthermore, targeting the anthrax toxins could increase the length of which treatment may be administered. A combination treatment that targets both bacterial growth and toxin production would be ideal and probably necessary for effectively combatting this armed bacterium.

## Abbreviations

ATP: Adenosine triphosphate
AVA: Anthrax vaccine absorbed
AVP: Anthrax vaccine precipitated
BID: Twice a day
CA: Cutaneous anthrax
CAD: Canadian dollars
CaM: Calmodulin
cAMP-3: 5′-cyclic adenosine monophosphate
cfu: Colony forming units
CMG-2: Capillary morphogenesis protein-2
EF: Edema factor
ET: Edema toxin
ETEC: Enterotoxinogenic E.coli
FDA: Food and drug administration
GA: Gastrointestinal anthrax
i.v: Intravenously
IA: Inhalational anthrax
IC_50_: Half maximal inhibitory concentration
IP: Intraperitoneal
IPDM: In vitro hollow fiber pharmacodynamic model
IαIp: Inter-α Inhibitor protein
kD: Dissociation constant
kDa: Kilodalton
LF: Lethal factor
LFI: Lethal factor inhibitors
LT: Lethal toxin
mAB: Monoclonal antibody
MAPKKs: Mitogen-activated protein kinase kinases
MIC: Minimum inhibitory concentration
MPI: Modified peptidomimetic inhibitors
MRSA: Methicillin-resistant *Staphylococcus aureus*
NHP: Non-human primates
PA: Protective antigen
PA20: 20 kDa PA
PA63: 63 kDa PA
PA83: 83 kDa PA
PBS: Phosphate-buffered saline
QD: Every day
SC: Subcutaneous
TEM-8: Tumor endothelial marker 8
UK: United Kingdom
USA: United States
